# Radio Frequency Magnetron Sputtering Deposition of TiO_2_ Thin Films and Their Perovskite Solar Cell Applications

**DOI:** 10.1038/srep17684

**Published:** 2015-12-03

**Authors:** Cong Chen, Yu Cheng, Qilin Dai, Hongwei Song

**Affiliations:** 1 State Key Laboratory on Integrated Optoelectronics, College of Electronic Science and Engineering, Jilin University, 2699 Qianjin Street, Changchun, 130012, People’s Republic of China

## Abstract

In this work, we report a physical deposition based, compact (cp) layer synthesis for planar heterojunction perovskite solar cells. Typical solution-based synthesis of cp layer for perovskite solar cells involves low-quality of thin films, high-temperature annealing, non-flexible devices, limitation of large-scale production and that the effects of the cp layer on carrier transport have not been fully understood. In this research, using radio frequency magnetron sputtering (RFMS), TiO_2_ cp layers were fabricated and the thickness could be controlled by deposition time; CH_3_NH_3_PbI_3_ films were prepared by evaporation & immersion (E & I) method, in which PbI_2_ films made by thermal evaporation technique were immersed in CH_3_NH_3_I solution. The devices exhibit power conversion efficiency (*PCE*) of 12.1% and the photovoltaic performance can maintain 77% of its initial *PCE* after 1440 h. The method developed in this study has the capability of fabricating large active area devices (40 × 40 mm^2^) showing a promising *PCE* of 4.8%. Low temperature and flexible devices were realized and a *PCE* of 8.9% was obtained on the PET/ITO substrates. These approaches could be used in thin film based solar cells which require high-quality films leading to reduced fabrication cost and improved device performance.

Methylammonium lead iodide (CH_3_NH_3_PbI_3_) perovskite based solar cells have been extensively studied due to the great potential to reduce the dependencies on fossil energy. It is reported that the perovskite solar cells achieve remarkably high efficiency of 19% for both mesoporous metal oxide scaffolds and planar heterojunction architectures because CH_3_NH_3_PbI_3_(MAPbI_3_), as light harvesting material, offers all the desirable characteristics such as large absorption coefficient, high charge carrier mobility and long diffusion length^1^,^2^,^3^. Planar architecture has been developed due to the enhanced device flexibility and multijunction construction application^4^. TCO/cp semiconductors oxide/perovskite materials/HTM/cathode is the standard configuration for planar heterojunction perovskite solar cells. The cp layer and CH_3_NH_3_PbI_3_ layer are indispensable in a perovskite solar cell, and their high quality directly plays an important role in reducing the structural and electronic defects in the films which can affect the device performance significantly. For the cp layer preparation, Spray pyrolysis and spin-coating precursor solution methods are two typical methods to prepare electron collecting cp layer for planar heterojunction perovskite solar cells. However, these two methods are also associated with certain disadvantages. First, the cp layer obtained by these two methods is not high-quality condensed layer. Second, it is difficult to control the cp-layer thickness for these two methods. Third, they involve high temperature preparation and non-flexible devices, which limits the applications of perovskite solar cells. In addition, spin coating technique is not suitable for commercial large-scale production of solar cells. Therefore, the investigation of cp layer preparation methods that involve high-quality condensed layer, easy control of thin film growth, low temperature preparation and flexible devices is necessary and will provide important insight on these issues. Atomic layer deposition (ALD) has been used to fabricate cp layers for perovskite solar cells, but ALD requires a relatively long time and high-cost for the thin film preparation^5^. For the CH_3_NH_3_PbI_3_ thin film synthesis, typically, the deposition of MAPbI_3_ perovskite thin films is accomplished using a one-step or two-step solution-processing method. However, it is difficult to control perovskite crystallinity and film uniformity due to the annealing process, which is unavoidable for the one-step solution-processing method. In general, the two-step method offers better control of the perovskite morphology compared with the one-step method. However, the spin-coating technique in the two-step method is related with low quality of films and limitation of large-scale production^6^. CH_3_NH_3_PbI_3_ thin film synthesis approach with a big achievement, which is based on a dual-source evaporation technique, has been developed for planar heterojunction perovskite solar cells^7^. However, this method requires special lab equipment.

In this work, we report for the first time a high-quality, easy-control, physical-deposition approach, which allows low temperature preparation for cp layer synthesis and the fabrication of large area and flexible devices for perovskite solar cell applications, which is based on RFMS technique. RFMS is a low-cost method for preparing semiconductor and metal thin films on various substrates, and it is particularly suitable for the growth of nanostructured films^8^,^9^,^10^. For CH_3_NH_3_PbI_3_ thin film preparation, we also modified the traditional two-step solution-processing method by E&I approach, instead of spin-coating, in which PbI_2_ thin films were prepared via thermal evaporation followed by immersing the PbI_2_ thin films in CH_3_NH_3_I solution. This E&I approach with great film reproducibility overcomes the problems of traditional one-step and two-step methods including incomplete conversion and uncontrolled growth of CH_3_NH_3_PbI_3_ thin films, and it is suited for large-scale production of solar cells. In addition, considering the rapid energy payback time of photovoltaic device^11^, RFMS technique and E&I method could be one alternative way to realize large area, low cost and commercialized devices.

In this research, planar heterojunction perovskite solar cells based on cp TiO_2_ electron transport layer and MAPbI_3_ thin film by RFMS combined with E & I method were fabricated and their performance was characterized. The best PCE of 12.1% with an open-circuit voltage (*V*_*OC*_) of 1.09 V was achieved. An average *PCE* of 10.9% was obtained and can maintain 77% of its initial *PCE* after 1440 h in this work. In addition, we also demonstrate that good performance flexible perovskite solar cells (*PCE* = 8.9%) on polyethylene terephthalate/indium-Tin Oxide (PET/ITO) substrate and large active area devices (40 × 40 mm2, *PCE* = 4.8%) can be obtained by the reported method in this paper.

## Results and Discussion

### Structural characterization of perovskite solar cells

**Figure 1:**
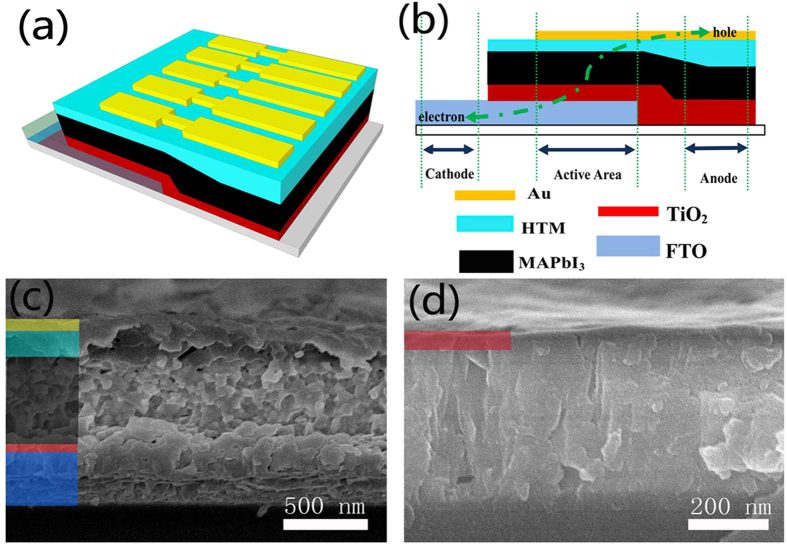
(**a**) Schematic illustration of the solar cells. (**b**) Schematic architecture of the investigated devices. (**c**) Cross-sectional SEM image of a typical perovskite solar cell by RFMS combined with E & I method. (**d**) Detailed view of the cp TiO_2_ layer (~60 nm).

Figure 1(a,b) show the typical three-dimensional structure and the schematic architecture of our perovskite solar cells, respectively. It consists of fluorine-doped tin oxide (FTO) transparent conductive anode, cp TiO_2_ electron transport layer, MAPbI_3_ active layer, 2,2′,7,7′-tetrakis-(N,N-di-p-methoxyphenylamine)-9,9′-spirobifluorene (spiro-oMeTAD) hole transport layer and Au cathode. The pathways of holes and electrons are shown in the schematic architecture. Figure 1(c) shows the cross-sectional scanning electronic microscopy (SEM) image of a well-constructed perovskite solar cell fabricated using RFMS combined with E & I method. The thicknesses of the MAPbI_3_, HTM, and gold layer are 750, 230 and 100 nm respectively. From Figure 1(c), well-defined infiltration boundaries of MAPbI_3_/ HTM can be observed clearly, and no pores/voids are visible even on the nanoscale. The interface between the MAPbI_3_ and HTM layers shows the effective combination of the two layers for heterojunction structure. Perovskite material and hole transport layer material have very uniform distribution, and such a dense structure will contribute to efficient light absorption and charge transport. Figure 1(d) shows the interface between TiO_2_ and FTO manifesting the infiltration of TiO_2_ into the FTO layer. A detailed view of the cp TiO_2_ electron transport layer with high uniformity shows the cp-layer thickness is approximately 60 nm, which is almost the same as the TiO_2_ commonly used in perovskite solar cells according to the literature^12^.

### The influence of TiO_2_ layer prepared by RFMS on device performance

**Figure 2:**
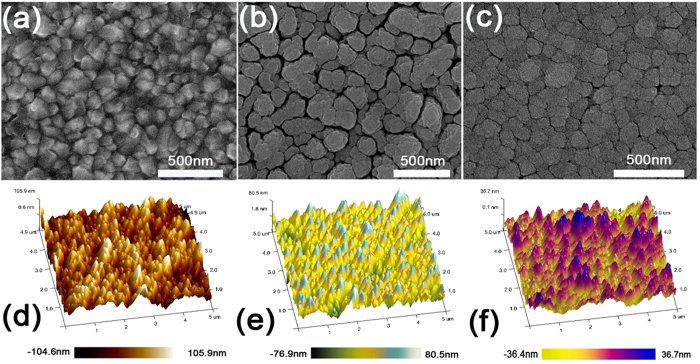
(**a**) and (**d**) show the SEM and AFM images of a bare FTO surface respectively. (**b**,**e**) show the SEM and AFM images of the surface of TiO_2_ on FTO with 15 min deposition respectively (**c**,**f**) show the SEM and AFM images of the surface of TiO_2_ on FTO with 30 min deposition respectively.

To study the effect of deposition time on the TiO_2_ film surface, the coating of TiO_2_ on FTO substrate was carried out using different sputtering time of 15, 30 and 60 min. Figure 2(a,d) show the SEM and AFM images of top surface of a bare FTO respectively. In Figure 2(a), uneven surface of commercial bare FTO can be observed, which has been reported by Masuda *et al.*^13^. The uneven surface can significantly affect the uniformity of the cp layer prepared by spray pyrolysis or spin-coating with precursor solution due to the diffusibility and surface tension of the liquid. The AFM image (Fig. 2(d)) of the bare FTO shows the depth changing from -104.6 to 105.9 nm. Figure 2(b) shows the surface morphology of TiO_2_ film prepared with 15 min sputtering. It can be observed that the surface uniformity has been improved to some extent which can be confirmed by the depth (−76.9 ~ 80.5 nm) shown in Fig. 2(e). However, there are still some relative large gaps caused by the uneven substrates. Fig. 2(c,f) show the SEM and AFM images of the surface morphology of TiO_2_ film respectively (sputtering time: 30 min). The film depth ranging from −36.4 nm to 36.7 nm indicates significant improved surface uniformity (Fig. 2(f)). The roughness of the films fabricated by RFMS is relatively low compared to other solution based films. All the samples were deposited under the same condition expect the sputtering time. The cp-layer thickness is increased with increasing sputtering time.

Recent studies showed that the cp-layer thickness had significant impact on the device performance of cp-TiO_2_/CH_3_NH_3_PbI_3_ planar heterojunction solar cells. The film thickness was controlled by varying the sputtering time from 0 to 60 min (0, 15, 20, 25, 30, 35, 40, 50 and 60 min) to investigate the influence of the thickness-dependent TiO_2_ on the device performance. Because some deposited TiO_2_ films are extremely thin within a few nanometers, the thicknesses are estimated by the film thickness monitor set in RFMS chamber.

We also fabricated the devices without the cp TiO_2_ electron transport layer. The device performance was characterized by current density (*J*)-voltage (*V*) measurements under simulated AM 1.5 G (100 mW/cm^2^) solar irradiation in the air. The device without the cp TiO_2_ electron transport layer (sputtering time = 0) still exhibits photovoltaic performance of ~4.3% (*J*_*sc*_ = 13.6 mA/cm^2^, *V*_*oc*_ = 0.93 V and *FF* = 34%). The *J*_sc_ and *V*_*oc*_ values are typical for perovskite solar cells based on TiO_2_ and spiro-OMeTAD.

**Figure 3:**
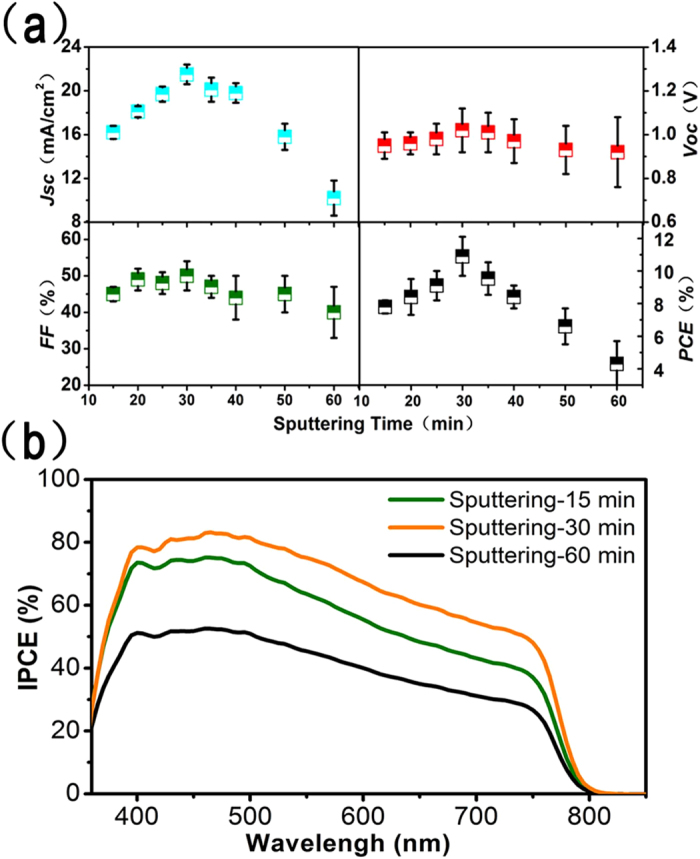
(**a**) shows the photovoltaic parameters of the devices based on different sputtering time. 12 solar cells were repeated and measured for each sputtering time. (**b**) IPCE spectra of perovskite solar cells based on three typical sputtering time.

The photovoltaic parameters (*J*_*sc*_, *V*_*oc*_, *FF* and *PCE*) of the devices based on different cp TiO_2_ layers with different sputtering time are plotted and summarized in Fig. 3(a) and Table 1. When the cp TiO_2_ electron transport layer was introduced into the perovskite solar cells, the device performance was significantly enhanced compared to that without cp layer. The 15 min sputtered TiO_2_ film (~22 nm) based perovskite cell shows a *PCE* of 7.8% with a *J*_*sc*_ of 16.1 mA /cm^2^, a *FF* of 51% and a *V*_*oc*_ of 0.95 mV. *J*_sc_, *V*_*oc*_*, FF* and *PCE* increase to 20.6 mA /cm^2^, 1.09 V, 54% and 12.1% respectively as the sputtering time increases to 30 min. In general, the devices exhibit *J*_*sc*_ = 21.5 ± 0.9 mA/cm^2^, *V*_*oc*_ = 1.02 ± 0.1 V, *FF* = 50  ± 4%, and the resulting *PCE* = 10.9 ± 1.2%. The *J*_*sc*_ increased with increasing cp-layer thickness, as the sputtering time is increased from 15 to 30 min. This can be attributed to the improved electron collection and effective hole blocking caused by the inserted TiO_2_ layer. However, with increasing time from 35 to 60 min corresponding to the thickness from 80 to 200 nm, the decreased device performance is observed due to lower electron transport in thicker cp layers and longer transmission distance leading to increased recombination rates. This can also be confirmed from the apparent decrease of *FF* parameters. Incident photon-to-current conversion efficiency (IPCE) measurement was also carried out for the devices based on TiO_2_ with different thicknesses. Figure 3(b) shows the typical IPCE spectra of perovskite solar cells, which is consistent with those in previous papers published^14^. After integrating the product of AM 1.5 G photon flux with the IPCE spectrum, the calculated *J*_*sc*_ is found to be 20.9 mA/cm^2^ which is in good agreement with measured *J*_*sc*_ value (21.5 ± 0.9 mA/cm^2^) obtained for the device with sputtering time of 30 min. The IPCE spectra follow the same trend with *PCE* obtained for different sputtering time. So the best device performance can be achieved when the optimum cp-layer thickness is employed. In addition, these results also indicate that n-type cp semiconductor layer plays a significant role in both electron transport and hole blocking within perovskite solar cells.

### Performance of the devices based on MAPbI_3_ perovskite synthesized by E & I

In planar heterojunction architecture, the MAPbI_3_ perovskite film, as a light-absorbing layer, is simply sandwiched between two charge selective extraction contacts and the device performance can be significantly affected by the quality and the coverage of MAPbI_3_ perovskite film. Herein, we prepared MAPbI_3_ perovskite film by an improved two-step approach (E & I).This is an improved two-step process in which PbI_2_ was evaporated to form a PbI_2_ film in the first step rather than spin-coating a PbI_2_ precursor in DMF solution^15^. This process can also avoid the extremely high reaction rate of lead halide perovskite film often observed in the co-deposition process^7^. This homogeneous PbI_2_ film acts as a superior framework and “nucleation” centers for the MAPbI_3_ formation with large crystals and regular morphology.

Evaporation is the deposition of lead iodide film by thermal evaporation method which is commonly used to prepare Au or Ag electrode. The evaporation temperature of PbI_2_ (~954 °C) is lower than that of Au (2807 °C) or Ag (2210 °C) under standard atmospheric conditions, and can be evaporated easily in vacuum environment. Immersion process, the perovskite material is formed, has dramatic influences on the crystallinity of MAPbI_3_ and internal structure of perovskite solar cells. Here, we systematically analyze the effects of conversion from PbI_2_ to MAPbI_3_ in immersion process on perovskite solar cell performance. Our results show that the suitable immersing time is critical to obtain full conversion from PbI_2_ to MAPbI_3_ to achieve optimized photocurrent and excellent photovoltaic performance.

**Figure 4:**
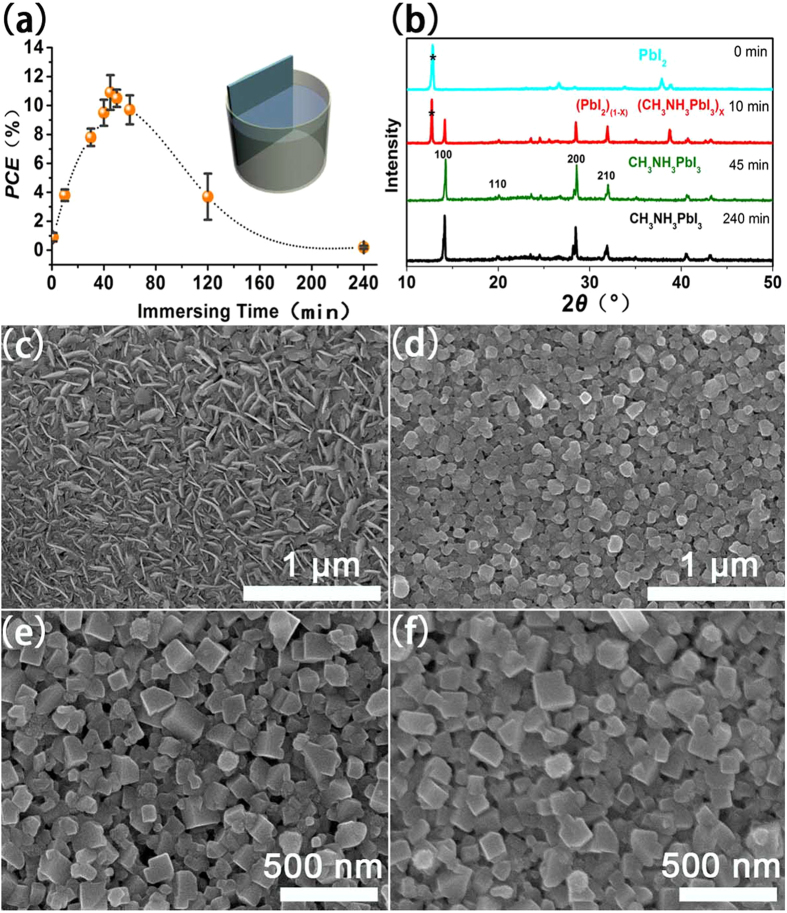
(**a**) The *PCE* of the devices as a function of immersing time. 12 solar cells were repeated and measured for each immersing time; (**b**) XRD patterns of PbI_2_ film with different immersing time in MAI solution; (**c**) SEM image of the PbI_2_ film deposited by thermal evaporation on Glass/FTO/cp-TiO_2_ substrate without immersing in MAI; (**d**) SEM image of the product obtained by immersing PbI_2_ film in CH_3_NH_3_I for 10 min; (**e**) SEM image of MAPbI_3_ layer prepared by immersing PbI_2_ film in MAI for 45 min; (**f**) SEM image of MAPbI_3_ layer fabricated with immersing PbI_2_ film in MAI for 240 min.

Figure 4(a) shows the *J-V* curves of different devices based on different immersing time of PbI_2_ in MAI. In order to monitor the conversion of the PbI_2_ phase to the perovskite phase, time-dependent X-ray Diffraction (XRD) (Fig. 4(b)) and corresponding SEM measurements (Fig. 4(c–f)) for different immersing time were also conducted. During the reaction process, it can be observed that the appearance and evolution of MAIPbI_3_ structure can be confirmed from XRD results and SEM images, which is consistent with literature results about cubic (*Pm3m*) phase of the perovskite MAIPbI_3_^16^,^17^,^18^. When the immersing time is increased from 0 min to 240 min, the short-circuit photocurrent density first increases and then decreases. In Fig. 4(b), the *2θ* peaks at 12.56^°^ (Fig. 4(b), marked with *) can be assigned to PbI_2_ as the immersing time is 0~10 min indicating the presence of unconverted PbI_2_. From Fig. 4(c), the thermal-evaporation PbI_2_ shows a stacked nanosheet structure which exhibits much larger surface area than traditional film to fully contact with the MAI in isopropanol solution. The changes in the crystal structure of the material compared with the original PbI_2_ film, suggests that E & I method promotes a rearrangement of PbI_2_ in CH_3_NH_3_I solution via intensive diffusion during film growth. One XRD peak (14.2^°^) is observed corresponding to the perovskite phase as the immersing time is 10 min (Fig. 4(b)). Simultaneously, some nanocrystals can be observed in Fig. 4(d) for the 10 min immersing time due to perovskite material formed on the surface of the film. The photovoltaic performance shows a rising trend as the immersing time is increased from 0 to 45 min due to the improved photocurrent caused by the more light absorber formed during the immersing process. The rising *PCE* trend from 0~45 min can also be concluded that the residual PbI_2_ layer in films that are not fully converted inhibits charge transfer from the perovskite to the cp TiO_2_ electron transport layer. PbI_2_ XRD peaks disappear (Fig. 4(b)), and larger nanocrystals with the size of ~150 nm can be observed (Fig. 4(e)) as immersing time is increased to 45 min, which indicates that the reaction of CH_3_NH_3_I and PbI_2_ is complete. Meanwhile, the *PCE* reaches the highest value which also confirms the complete perovskite conversion during the immersion process. Such a behavior has been observed previously for this family of materials and is currently under intensive investigation by several research groups^19^,^20^. *FF* parameters of these devices decrease significantly with increasing immersing time from 60 min (*FF* = 51%) to 240 min (*FF* = 25.5%). The same trend can be observed for the conversion efficiency, decreasing from 10.9% for 45 min to 0.21% for 240 min. To better understand the influence of longer immersing time (>60 min) on the perovskite film growth, XRD and SEM of the sample with 240 min immersing are measured and shown in Fig. 4(b,f). It is clear that there is no apparent difference in crystallinity, crystal size and surface morphology, which indicates that longer immersing time has limited influence on MAPbI_3_ light absorbing films. Similar results were also obtained in other work^21^. But the cross-sectional images of the MAPbI_3_ films on TiO_2_-coated FTO in Figure S1(a)~2(c) can explain the decreased device performance for the longer immersing time. It can be observed that the MAPbI_3_ films peel off from Glass/FTO/cp-TiO_2_ for the samples with immersing time 120 and 240 min (Figure S1(b,c)) in comparison with that of 45 min in Figure S1(a). It is possible that the surface tension of the MAI solution affects the stability of the film leading to decreased device performance for the longer immersing time. Thus the conversion extent of the lead iodide film determined by immersing time is critical for optimizing device performance. (PbI_2_)_1-x_ (MAPbI_3_)_x_ films are not converted completely when the immersing time is less than 10 min, and longer immersing time (more than 60 min) leads to fracture of the device. The transformation process of PbI_2_ into CH_3_NH_3_PbI_3_ has been investigated by Lang *et al.*^22^. They proposed that the diffusion of Pb allows the formation of CH_3_NH_3_PbI_3_ from stacked PbI_2_ and CH_3_NH_3_I by inter-diffusion, and the perovskite crystallites at the surface are associated with the diffusion. We believe that similar mechanism happened to our system. The transformation time in their study is shorter from PbI_2_ to CH_3_NH_3_PbI_3_ compared to the reaction time in this work. However, PbI_2_ was dissolved in Dimethylformamide solution and infiltrated into np-TiO_2_ mesoporous structure by spin coating in their work. That may lead to shorter transformation time compared to PbI_2_ dense films prepared by thermal evaporation in this work.

### Anomalous hysteresis for the best efficient device

Anomalous Hysteresis has been studied to determine the performance of perovskite solar cells^2^,^23^,^24^. A series of the relevant tests have been carried out for the device which exhibits the best PCE of 12.1% in this study. The influence of scanning conditions on the J-V curves is shown in Figure S2. The testing device was measured under simulated AM1.5 with different scan rates from 2 to 0.04 V/s from forward bias to short circuit (FB-SC). As the scan rate is 2 V/s, the testing device shows better performance (*PCE* = 12.7%). However, by slowing down the scan rate, the hysteresis appears. When the scan rate is reduced to 0.04 V/s, PCE of the device decreases to 11.5%. When the scanning rate is 0.6 ~ 0.4 V/s, the device exhibits relatively stable *PCE* of ~12%.

Scanning directions have a certain influence on the performance of the devices. When the device was test in SC-FB with a scan rate of 0.5 V/s, the *PCE* was 11.7% with *J*_*sc*_ = 19.8 m A/cm^−2^, *V*_*oc*_ = 1.0 V, *FF* = 59% which is shown in Figure S3. This phenomenon is more obvious in other published results^24^,^25^. The reasons can be concluded as follows: i) The perovskite absorber CH_3_NH_3_PbI_3_ may have some surface defects which could act as traps for electrons and holes and fill under forward bias working conditions. Then the trapping and detrapping times are likely to vary with architecture and processing^2^. ii) The organometal trihalide perovskites have been observed to possess ferroelectric properties. Upon applied bias, a slow polarization of CH_3_NH_3_PbI_3_ may occur, which could be responsible for the hysteresis^16^,^23^.

### Flexible perovskite solar cells

**Figure 5:**
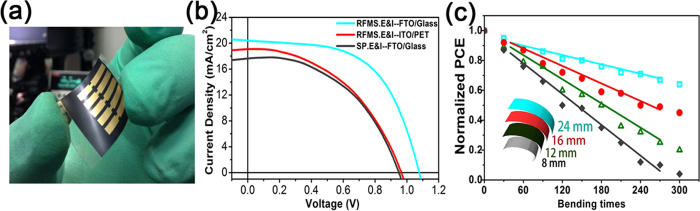
(**a**) Photograph of flexible devices in this study. (**b**) *J-V* curves of the devices on Glass/FTO and PET/ ITO substrates based on RFMS technique compared to that of the device on Glass/FTO based on spin-coating approach. (**c**) Overall performance changes with the increasing times of bending cycles. All the PCE data were normalized to describe the device performance trends.

Cp TiO_2_ electron transport layers were also deposited on PET/ITO substrate by RFMS technique to fabricate flexible perovskite solar cells with a configuration of PET/ITO/TiO_2_/MAPbI_3_/spiro-oMeTAD/Au in this study. The experimental conditions and procedure of the flexible devices are the same as those of the devices on Glass/FTO. Figure 5(a) shows a photograph of the flexible devices prepared by the method developed in this work. Figure 5(b) shows the *J–V* curves of three kinds of perovskite solar cells prepared on different substrates by different methods. The average performance of the flexible device (red line in Fig. 5(b), *J*_*sc*_ = 19.0 m A/cm^−2^, *V*_*oc*_ = 0.96 V, *FF* = 48% and *PCE* = 8.9%) is not as good as that of the device on Glass/FTO (blue line in Fig. 5(b), *J*_*sc*_ = 20.6 mA/cm^−2^, *V*_*oc*_ = 1.09 V, *FF* = 54% and *PCE* = 12.1%) which was prepared with the same experimental parameters. The reduced device performance of the flexible solar cell can be attributed to large sheet resistance of PET/ITO (60 Ω/sq) and the low light transmittance (78%) compared to those of Glass/FTO (7Ω/sq, 92%). However, the *PCE* of 8.9% for the flexible device is comparable to the reported results for flexible solar cells^26^,^27^,^28^.

A device with the same fabrication process except that the TiO_2_ layer prepared by spin-coating method (annealed at 500 °C for 30 min) exhibits a lower *PCE* of 7.9% shown in Fig. 5(b) (black line) compared to that of the RFMS prepared TiO_2_ (12.1%). This indicates the RFMS is a very promising method to fabricate high efficiency perovskite solar cells compared to conventional spin-coating method in which the desired cp layer with uniform thickness cannot be easily prepared due to the uneven surface of the commercial FTO (Fig. 3(a)) and the flow ability of spin-coating precursor. Thus better solar cell performance can be obtained for the devices based on the uniform films prepared by RFMS combined with E & I method compared with those based on conventional spin-coating method.

**Figure 6:**
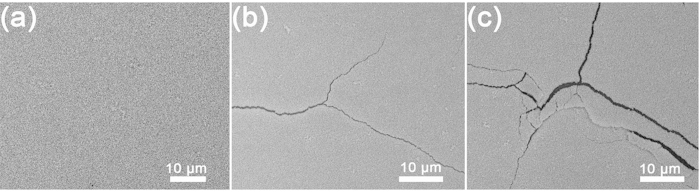
(**a**) SEM image of perovskite MAPbI_3_ surface without bending. (**b**) SEM image of perovskite MAPbI_3_ after bending with the angle of 60° for 300 cycles. (**c**) SEM image of perovskite MAPbI_3_ after bending with the angle of 180for 300 cycles.

The device performance after mechanical bending was studied for the flexible devices. In Fig. 6(c), the flexible solar cells were bent over a roll with radii of 24 mm, 16 mm, 12 mm and 8 mm corresponding to the angles of 60°, 90°, 120° and 180° respectively. The specific bending process is described in the experimental section. Normalized *PCE*s of perovskite solar cells after bending with different radii and times were presented in Table S1. It can be observed that the *PCE* are estimated to be 8.5%, 8.2% and 7.9% after 30 bending cycles for the angles of 60°, 90° and 120° respectively, which is much higher than the reported results^27^. The *PCE* remains 7.7% after 30 bending cycles for the angle of 180° is still comparable to the data from the literature^27^. The performance of all the devices can be evaluated up to 300 bending cycles (600 times) indicating that our devices tolerate repeated mechanical deformation. The *PCE* with a bending angle of 60^o^, which remains 5.7% after 300 cycles bending, is more stable than other bending angles. This can be understood by the less deformation caused by smaller bending angle.

The reason for device performance degradation is the perovskite material surface structure changes with the bending as shown in Fig. 6. Figure 6(a) shows smooth surface of perovskite MAPbI_3_ without bending. Minor fracture surface occurred after bending with the angle of 60°for 300 cycles (Fig. 6(b)). Relatively large and crushed fracture happened after bending with the angle of 180°for 300 cycles in Fig. 6(c).

### Large active area perovskite solar cells

**Figure 7:**
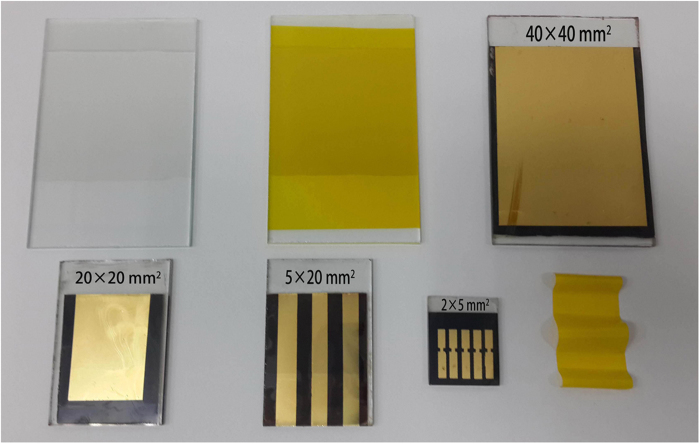
Photograph of perovskite solar cells with different active areas. The above three samples are RMFS-TiO_2_/FTO/Glass, E & I-PbI_2_ /RMFS-TiO_2_/FTO/Glass and Au/ spiro-OMeTAD/MAPbI_3_/ RMFS-TiO_2_/FTO/Glass with an active area of 40 × 40 mm^2^. The following represents three active areas of 20 × 20 mm^2^, 5 × 20 mm^2^ and 2 × 5 mm^2^, respectively. The last one is PbI_2_ is deposited on a slim plastic material (48 μm) by the method of thermal evaporation.

The reported high efficiency perovskite solar cells are based on small active areas (0.1 mm^2^~1 cm^2^), which is not suitable for practical application^29^. Research on large area perovskite solar cells is necessary and has not been addressed in full detail, which is related to the preparation process of perovskite solar cells, including the high-temperature annealing and spin-coating technique. Because of the potential in low costs and high performance, many considerable efforts are being increasingly undertaken to enable a commercial upscaling of this new type of solar cell. We have tried to address the problems associated with large active layer perovskite solar cell preparation. The devices were prepared by the similar method described in the experimental section except that different active areas were employed. The photograph of perovskite solar cells with different active areas can be found in Fig. 7. *J-V* curves of samples with an active area of 40 × 40 mm^2^, 20 × 20 mm^2^, 5 × 20 mm^2^ and 2 × 5 mm^2^ are shown in Figure S3, clearly. The devices based on 40 × 40 mm^2^ active area exhibit *J*_*sc*_ of 6~11 mA/cm^2^, *V*_*oc*_ of 0.8 ~ 1.2 V, *FF* of 40 ~ 60% and *PCE* of 4.2 ~ 4.9%. Enhanced device performance is observed as the active area decreases. *PCE* of ~5.6% and ~7.7% were obtained for 20 × 20 mm^2^ and 5 × 20 mm^2^ devices respectively. The *V*_*oc*_ values of large active area devices are similar to those of 0.1 cm^2^ perovskite solar cells indicating that that size of the active area has no effect on the *V*_*oc*_ values. The *J*_*sc*_ and *FF* parameters gradually decrease with increasing active area, which can be explained by more surface defects are involved for larger active area.

### The long-term stability of perovskite solar cells

**Figure 8:**
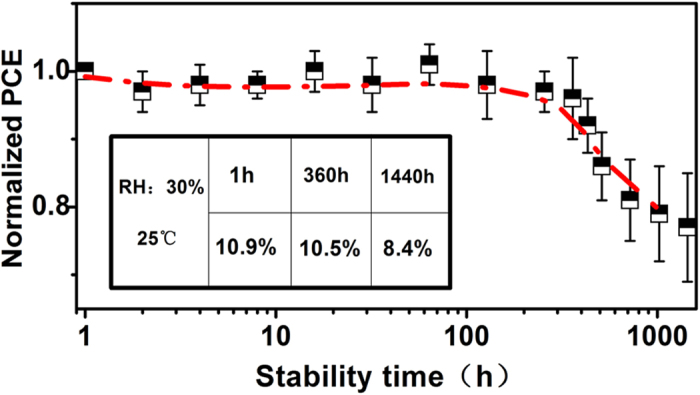
Time-dependent *PCE* of 15 representative devices. The table inset shows the average *PCEs* of the devices kept for 1 h, 360 h and 1440 h in the ambient conditions.

The long-term stability of perovskite solar cells is another crucial issue for the commercialization of perovskite solar cells, and the degradation mechanism of perovskite solar cells is not clearly understood. However, only a few work paid attention to the stability of perovskite solar cells, in which organic materials (PCBM, PEDOT:PSS, *etc*) were introduced. The organic materials decompose easily caused by oxygen and water in the air^30^. We believe that reduction of the structural configuration and improvement of the film quality are two effective methods to obtain high efficiency solar cells with long-term stability. RFMS combined with E & I method provides a new preparation process for perovskite solar cells in which high-quality films are used. The cells were kept open circuit under illumination of 14 W fluorescent lamps with a distance of 2.5 m to the light source in ambient air over 60 days (1440 h) with an unsealed dish. Figure 8 presents the time-dependent *PCE* for the devices based on RFMS combined with E & I method. The devices exhibit excellent stability with average PCE of ~10.5% after 360 h exposure in ambient air with relative humidity of 30% and temperature of 25 °C. Although the decline of *PCE* occurred after 1440 h testing, the stability of our devices is comparable to the perovskite solar cells based on organic carrier-transporting materials^31^,^32^.

In this work, the *FFs* values of all devices exhibit under 60% which is lower than those in some recent works^33^,^34^,^35^. It is reported that the *FF* values of perovskite solar cells are sensitive to the composition and the electron-hole extraction layer thickness^36^. Thus we attribute the lower *FF*s values to the composition and spiro-oMeTAD layer thickness.

## Conclusion

In summary, we developed a novel strategy to fabricate planar heterojunction perovskite solar cells by employing TiO_2_ cp layers made by RFMS method and perovskite layers obtained by E&I method. The physical method with easy control of thin film growth requires a much shorter time and overcomes low-quality of thin films, high-temperature annealing, non-flexible devices and limitation of large-scale production which are involved in the traditional methods. The best device on FTO obtained in this study exhibits an average efficiency of 10.9% and the best performance of 12.1% which is based on ~60 nm TiO_2_ cp layer. A of 8.9% was achieved for the flexible solar cells on PET/ITO, which is comparable with traditional methods. The photovoltaic performance of the flexible solar cells still can be evaluated up to 300 cycles bending indicating the suitability for roll to roll processing. Additionally, large active area (40 × 40 mm^2^) devices were also fabricated via this new strategy and a *PCE* of 4.8% was obtained. This RFMS combined with E & I method could be utilized in other thin film-based solar cells, and it also could be used to investigate the fundamental issues and enhance photovoltaic performance.

## Methods

### RFMS technique

RFMS is accomplished by the collision of the incident Ar^+^ ions and the target (semiconductor oxide). In this process, with an electric field E, some ultrafast electrons collide with argon atoms when flying to substrate. Ar atoms produce positive ions (Ar^+^) and new electrons (e^−^), and then the Ar^+^ ions as the incident particles experience in scattering target and colliding with the target. A part of the momentum of incident (Ar^+^) ions is delivered to the target atoms, and these target atoms and other target atoms collide to form a cascade process. In this cascade process certain target atoms near the surface with sufficient momentum move away from the target to be sputtered toward substrate. It has fast-deposition and surface-cleaning characteristics. Specific schematic process can be found in Figure S4^10^,^37^.

### Specific procedure for the assembly of the perovskite solar cells

Process 1: Initially FTO was removed from regions under the anode contact by etching the FTO with 30% HCl and zinc powder. Substrates were then cleaned with deionized water, acetone, and methanol and finally treated under oxygen plasma for 10 min to remove the last traces of organic residues.

Process 2: Cp TiO_2_ electron transport layer was deposited onto Glass/FTO or PET/ITO substrates to obtain electron collecting cp layer by RFMS. The TiO_2_ target has a purity of 99.99% and is 52 mm in diameter and 5 mm in thickness. The distance between the target and the substrate was approximately 50 mm. Prior to deposition, the chamber was evacuated to a pressure of ~4 × 10^−4^ Pa. The RF sputtering processes were performed in pure Ar gas (99.999%). The gas flow rate of Ar was controlled by a mass-flow controller and the chamber was kept at a working pressure of 3.5 Pa. In order to clean the target surface, a pre-sputtering process was introduced for 10 min before the deposition. The flow rate of pure Ar was fixed at 32 sccm (standard cubic centimeter per minute) and the TiO_2_ target was sputtered with a sputtering power of 120 W. The film thickness can be adjusted by the sputtering time and monitored by the film-thickness meter which is mounted on the substrate stage in the chamber.

Process 3: The PbI_2_ film was deposited on the TiO_2_ cp layer to form FTO/TiO_2_/ PbI_2_. The PbI_2_ powders were used to fabricate PbI_2_ films via thermal evaporation process (evaporation electric current: 50 A; evaporation voltage: ~220 V; Chamber pressure: ~8 × 10^−4^ Pa). The PbI_2_ film thickness, which is detected by the film-thickness meter in the chamber, can be controlled by the evaporation time.

Process 4: Immersing the FTO/TiO_2_/PbI_2_ film in a solution of MAI in isopropanol (10 mg/ml) allows the formation of MAPbI_3_ through the reaction of PbI_2_ and MAI. The color of the film changed immediately from red-brown to dark brown, indicating the production of MAPbI_3_. When the reaction was finished, the devices were transferred into pure isopropanol solution to rinse off excess MAI and then kept 5 min at 70 °C for drying on hot plate.

Process 5: The HTM was deposited by spin-coating a solution of spiro-OMeTAD at 2000 rpm for 35 s in nitrogen atmosphere and left in a closed dry box for 25 min. The spiro-oMeTAD solution was prepared by adding 75 mg of spiro-oMeTAD, 30 μL of 4-tert-butylpyridine and 10 mg /m LiN(CF_3_SO_2_)_2_ N to 1 mL of chlorobenzene then stirred for 1 hour before spin-coating.

Process 6: Au electrode with a thickness 100 nm was deposited on the top of HTM via thermal evaporation in a vacuum chamber (9 × 10^−4^ Pa). An evaporation mask was used to define the areas of the devices, and the active area of each device was controlled to be 0.1 cm2 (2 × 5 mm^2^), 1 cm^2^ (5 × 20 mm^2^), 4 cm^2^ (20 × 20 mm^2^) and 16 cm^2^ (40 × 40 mm^2^).

### Raw chemicals and precursor preparation

All reagent grade chemicals were obtained commercially from Sigma-Aldrich. The TiO_2_ target was bought from China New Metal Company with 5N purity. MAI was prepared in house with the method described in literature^15^. In a typical procedure, Methylamine (CH_3_NH_2_) (13.5 mL, 40 wt% in aqueous solution) reacted with hydroiodic acid (HI) (15.0 mL, 57 wt% in water) in 250 ml round bottomed flask at 0 °C for 2 h with stirring. The solvent and excess CH_3_NH_2_ were removed using a rotary evaporator, and the initial MAI powders were obtained. The precipitate was then washed three times with diethyl ether, and then dried at 60 °C in a vacuum oven for 12 h.

### Measurements

Non-masked devices were tested under a Class A solar simulator (ABET Sun 2000) at AM1.5 and 100 m/cm^2^ illumination conditions calibrated with a reference Silicon cell (RERA Solutions RR-1002), using a Keithley 2400 as a source-meter in ambient condition without sealing for *J-V* measurements with 1500 voltage points from + 1.5 V to -1.5 V. IPCE was measured at AC mode under bias light using a IPCE system (PV measurement Inc.) with a computerized setup consisting of Solar Cell Quantum Efficiency — Solar-CellScan100. X-ray diffraction (XRD) was performed on an X-ray diffractometer (D8-Advance, Bruker, Germany) using Cu Ka1 radiation (l ¼ 1.5406 A˚) at step size/time of 0.02/1 s. The surface morphology of the films and cross-sections of the perovskite solar cells were characterized by a SIRION field-emission scanning electron microscope. The local roughness of the MAPbI_3_ thin films were characterized by atomic force microscopy (AFM; 5500, Agilent, Santa Clara, CA) operated in contact mode.

### Specific procedure for bending test

For the bending test, the cells were bended 300 cycles (600 times) on a cylinder with different radii. In every 1 cycle, the cell was flipped to experience both compression and extension stresses, and a *J-V* measurement was carried out every 30 cycles (60 times).

## Additional Information

**How to cite this article**: Chen, C. *et al.* Radio Frequency Magnetron Sputtering Deposition of TiO_2_ Thin Films and Their Perovskite Solar Cell Applications. *Sci. Rep.* **5**, 17684; doi: 10.1038/srep17684 (2015).

## Supplementary Material

Supporting Information

## Figures and Tables

**Table 1:** Photovoltaic parameters (*Jsc*, *Voc*, *FF* and *PCE*) of the devices based on different sputtering time.

Sputtering time (min)	*J*_*sc*_(mA/cm^2^)	*V*_*oc*_ (V)	*FF*(%)	*PCE*(%)
15	16.2 ± 0.6	0.95 ± 0.06	45 ± 2	7.8 ± 0.4
20	18.1 ± 0.5	0.96 ± 0.05	49 ± 3	8.4 ± 1.1
25	19.7 ± 0.7	0.98 ± 0.07	48 ± 3	9.1 ± 0.9
30	21.5 ± 0.9	1.02 ± 0.10	50 ± 4	10.9% ± 1.2
	20.6	1.09	54	12.1
35	20.1 ± 1.1	1.01 ± 0.09	47 ± 3	9.5 ± 1.0
40	19.8 ± 0.9	0.97 ± 0.1	44 ± 6	8.4 ± 0.7
50	15.8 ± 1.2	0.93 ± 0.11	45 ± 5	6.6 ± 1.1
60	10.2 ± 1.6	0.92 ± 0.08	45 ± 7	4.3 ± 1.4

## References

[b1] Docampo P., Ball J. M., Darwich M., Eperon G. E. & Snaith H. J. Efficient organometal trihalide perovskite planar-heterojunction solar cells on flexible polymer substrates. Nat. Commun. 4, 761 (2013). 10.1038/ncomms3761 24217714

[b2] Kim H.-S. *et al.* Mechanism of carrier accumulation in perovskite thin-absorber solar cells. Nat. Commun. 4, 242 (2013). 10.1038/ncomms3242 23900067

[b3] Leijtens T. *et al.* Overcoming ultraviolet light instability of sensitized TiO_2_ with meso-superstructured organometal tri-halide perovskite solar cells. Nat. Commun. 4, 885 (2013). 10.1038/ncomms3885 24301460

[b4] Chen Q. *et al.* Planar Heterojunction Perovskite Solar Cells via Vapor-Assisted Solution Process. J. Am. Chem. Soc. 136, 622–625 (2014). 24359486 10.1021/ja411509g

[b5] Di Giacomo F. *et al.* Flexible Perovskite Photovoltaic Modules and Solar Cells Based on Atomic Layer Deposited Compact Layers and UV-Irradiated TiO_2_ Scaffolds on Plastic Substrates. Adv. Energy Mater. 5, 1401808 (2015).

[b6] Yongzhen W. *et al.* Highly compact TiO_2_ layer for efficient hole-blocking in perovskite solar cells. Appl. Phys. Express. 7, 052301 (2014).

[b7] Liu M. Z., Johnston M. B. & Snaith H. J. Efficient planar heterojunction perovskite solar cells by vapour deposition. Nature. 501, 2509 (2013). 10.1038/nature12509 24025775

[b8] Liu Q. Q. *et al.* Effects of RF and pulsed DC sputtered TiO_2_ compact layer on the performance dye-sensitized solar cells. Surf. Coat. Tech. 231, 126–130 (2013).

[b9] Jeong J.-A. & Kim H.-K. Thickness effect of RF sputtered TiO_2_ passivating layer on the performance of dye-sensitized solar cells. Sol. Energ. Mat. Sol. C. 95, 344–348 (2011).

[b10] Kitano M., Funatsu K., Matsuoka M., Ueshima M. & Anpo M. Preparation of nitrogen-substituted TiO_2_ thin film photocatalysts by the radio frequency magnetron sputtering deposition method and their photocatalytic reactivity under visible light irradiation. J. Phys. Chem. B. 110, 25266–25272 (2006). 17165971 10.1021/jp064893e

[b11] Gong J., Darling S. B. & You F. Perovskite photovoltaics: life-cycle assessment of energy and environmental impacts. Energ. Environ. Sci. 8, 1953–1968 (2015).

[b12] Wojciechowski K., Saliba M., Leijtens T., Abate A. & Snaith H. J. Sub-150 C processed meso-superstructured perovskite solar cells with enhanced efficiency. Energ. Environ. Sci. 7, 1142–1147 (2014).

[b13] Masuda Y., Ohji T. & Kato K. Room-temperature synthesis of tin oxide nano-electrodes in aqueous solutions. Thin Solid Films 518, 850–852 (2009).

[b14] Lee J.-W., Seol D.-J., Cho A.-N. & Park N.-G. High-Efficiency Perovskite Solar Cells Based on the Black Polymorph of HC(NH_2_)_2_PbI_3_. Aav. Mater. 26, 4991–4998 (2014). 10.1002/adma.201401137 24923708

[b15] Burschka J. *et al.* Sequential deposition as a route to high-performance perovskite-sensitized solar cells. Nature. 499, 316 (2013). 23842493 10.1038/nature12340

[b16] Stoumpos C. C., Malliakas C. D. & Kanatzidis M. G. Semiconducting Tin and Lead Iodide Perovskites with Organic Cations: Phase Transitions, High Mobilities, and Near-Infrared Photoluminescent Properties. Inorg. Chem. 52, 9019–9038 (2013). 23834108 10.1021/ic401215x

[b17] Im J.-H., Jang I.-H., Pellet N., Grätzel M. & Park & N.-G. Growth of CH_3_NH_3_PbI_3_ cuboids with controlled size for high-efficiency perovskite solar cells. Nat. Nano. 9, 927–932 (2014). 10.1038/nnano.2014.181 25173829

[b18] Baikie T. *et al.* Synthesis and crystal chemistry of the hybrid perovskite (CH_3_NH_3_)PbI_3_ for solid-state sensitised solar cell applications. J. Mater. Chem. A. 1, 5628–5641 (2013).

[b19] Herman M. Jankovec M. & Topic M. Optimal I−V Curve Scan Time of Solar Cells and Modules in Light of Irradiance Level. Int. J. Photoenergy 2012, 151452-1−151452-11 (2012).

[b20] Frost J. M. *et al.* Atomistic Origins of High-Performance in Hybrid Halide Perovskite Solar Cells. Nano Lett. 14, 2584–2590 (2014). 24684284 10.1021/nl500390f PMC4022647

[b21] Docampo P. *et al.* Influence of the orientation of methylammonium lead iodide perovskite crystals on solar cell performance. Apl Mater. 2, 081508 (2014).

[b22] Lang F., Juma A., Somsongkul V., Dittrich T. & Arunchaiya M. Rutherford Backscattering Spectroscopy of Mass Transport by Transformation of PbI. Hybird Mater. 1, 52–61 (2014).

[b23] Snaith H. J. *et al.* Anomalous Hysteresis in Perovskite Solar Cells. J. Phys. Chem. Lett. 5, 1511–1515 (2014). 26270088 10.1021/jz500113x

[b24] Unger E. L. *et al.* Hysteresis and transient behavior in current-voltage measurements of hybrid-perovskite absorber solar cells. Energ. Environ. Sci. 7, 3690–3698 (2014).

[b25] Sanchez R. S. *et al.* Slow dynamic processes in lead halide perovskite solar cells. Characteristic times and hysteresis. J. Phys. Chem. Lett. 5, 2357–2363 (2014). 26279559 10.1021/jz5011187

[b26] Kaltenbrunner M. *et al.* Ultrathin and lightweight organic solar cells with high flexibility. Nat. Commun. 3, 770 (2012). 22473014 10.1038/ncomms1772 PMC3337988

[b27] Roldan-Carmona C. *et al.* Flexible high efficiency perovskite solar cells. Energ. Environ. Sci. 7, 994–997 (2014).

[b28] You J. *et al.* Low-Temperature Solution-Processed Perovskite Solar Cells with High Efficiency and Flexibility. Acs Nano. 8, 1674–1680 (2014). 24386933 10.1021/nn406020d

[b29] Jeon N. J. *et al.* Solvent engineering for high-performance inorganic-organic hybrid perovskite solar cells. Nat. Mater. 13, 897–903 (2014). 24997740 10.1038/nmat4014

[b30] Jørgensen M., Norrman K. & Krebs F. C. Stability/degradation of polymer solar cells. Sol. Energ. Mat. Sol. C. 92, 686–714 (2008).

[b31] Kim S.-H. *et al.* Fluorine-functionalized and simultaneously reduced graphene oxide as a novel hole transporting layer for highly efficient and stable organic photovoltaic cells. Nanoscale 6, 7183–7187 (2014). 24801948 10.1039/c4nr01038h

[b32] Edri E. *et al.* Why Lead Methylammonium Tri-Iodide Perovskite-Based Solar Cells Require a Mesoporous Electron Transporting Scaffold (but Not Necessarily a Hole Conductor). Nano Lett. 14, 1000–1004 (2014). 24475878 10.1021/nl404454h

[b33] Huang Y.-C. *et al.* Insight into Evolution, Processing and Performance of Multi-length-scale Structures in Planar Heterojunction Perovskite Solar Cells. Sci. Rep. 5, 13657 (2015). 26338280 10.1038/srep13657 PMC4559897

[b34] Jeon Y. J. *et al.* Planar heterojunction perovskite solar cells with superior reproducibility. Sci. Rep. 4, 7 (2014). 10.1038/srep06953 PMC4223662 25377945

[b35] Qin P. *et al.* A Novel Oligomer as a Hole Transporting Material for Efficient Perovskite Solar Cells. Adv. Energy Mater. 5, 1400980 (2015).

[b36] Wang Q. *et al.* Large fill-factor bilayer iodine perovskite solar cells fabricated by a low-temperature solution-process. Energ. Environ. Sci. 7, 2359–2365 (2014).

[b37] Kim K. K. *et al.* The grain size effects on the photoluminescence of ZnO/alpha-Al_2_O_3_ grown by radio-frequency magnetron sputtering. J. Appl. Phys. 87, 3573–3575 (2000).

