# Supplementary material for: Radio Frequency Magnetron Sputtering Deposition of TiO2 Thin Films and Their Perovskite Solar Cell Applications
Source: Sci Rep. 2015 Dec 3;5:17684. doi: 10.1038/srep17684 (PMC4668551; doi:10.1038/srep17684)
Supplement: Supporting Information [file srep17684-s1.doc]

**Supporting Information**

Radio Frequency Magnetron Sputtering Deposition of TiO_2_ Thin Films and Their Perovskite Solar Cell Applications

Cong Chen, Yu Cheng, Qilin Dai^*^ and Hongwei Song^*^


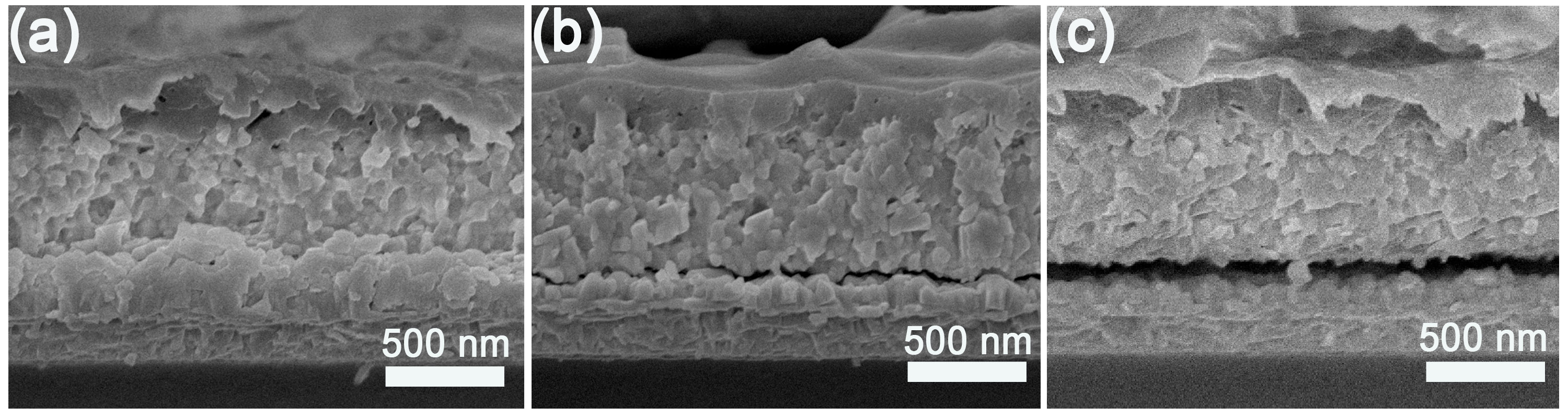


Figure S1. Cross-sectional SEM images of three typical immersion time ((a): 45 min, (b): 120 min and (c): 240 min)


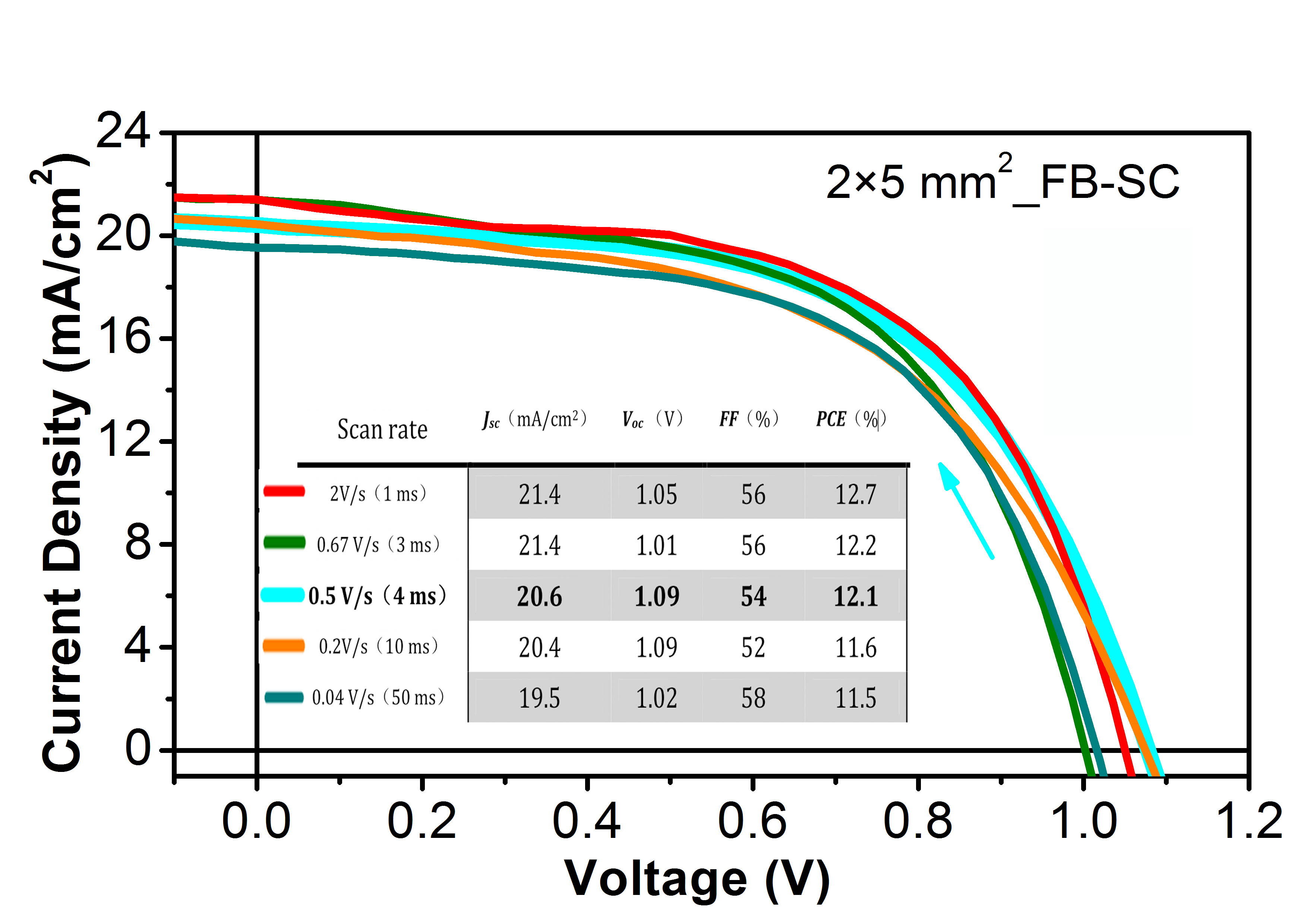


Figure S2. Comparision of the influence with different scanning conditions on Glass/FTO substrates based on RFMS technique. From forward bias to short circuit (FB-SC) *J-V* curves for the 2×5 mm^2^ device which exhibites best *PCE* at 12.1% with a scan rate of 0.5V/s which adopted in the whole experiment process.The device was also measured at a range of scan rates from 2 to 0.04 V/s. Different parameters of *J_sc_*,*V_oc_* and *FF* with different scan rates have been concluded in the inset figure.


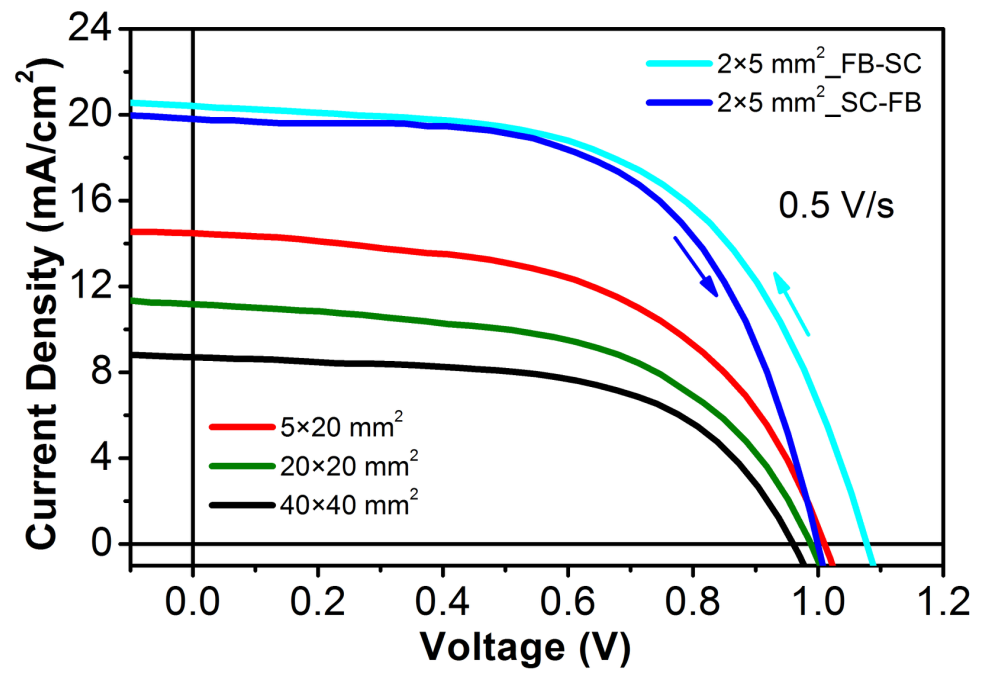


Figure S3. *J-V* curves(FB-SC) of samples with an active area of 40 × 40 mm^2^(black curve),20 × 20 mm^2^(Green curve), 5 × 20 mm^2^(red curve) and 2 × 5 mm^2^(cerulean curve) are shown clearly. All devices were prepared on Glass/FTO substrates by RFMS combined with E & I method. SC-FB *J-V* curve measured under simulated AM1.5 with an active area of 2 × 5 mm^2^ is exhibited with bule line.


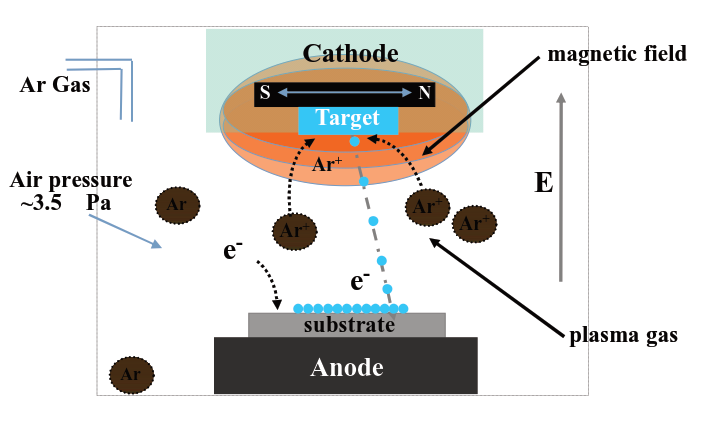


Figure S4. A schematic diagram of RF magnetron sputtering.

Table S1 (Normalized PCE of perovskite solar cells after bending with different radii and times.)

| **Bending radii**  **Bending times** | **24 mm（60°）** | **16 mm（90°）** | **12 mm （120°）** | **8mm（180°）** |
| --- | --- | --- | --- | --- |
| **0** | 1 | 1 | 1 | 1 |
| **30** | 0.95 | 0.92 | 0.89 | 0.87 |
| **60** | 0.88 | 0.87 | 0.79 | 0.76 |
| **90** | 0.86 | 0.78 | 0.76 | 0.66 |
| **120** | 0.81 | 0.72 | 0.60 | 0.50 |
| **150** | 0.80 | 0.68 | 0.57 | 0.48 |
| **180** | 0.76 | 0.59 | 0.50 | 0.35 |
| **210** | 0.72 | 0.58 | 0.36 | 0.25 |
| **240** | 0.71 | 0.50 | 0.32 | 0.12 |
| **270** | 0.67 | 0.49 | 0.25 | 0.10 |
| **300** | 0.64 | 0.45 | 0.20 | 0.04 |
